# Reference gene validation for quantification of gene expression during ovarian development of turbot (*Scophthalmus maximus*)

**DOI:** 10.1038/s41598-020-57633-3

**Published:** 2020-01-21

**Authors:** Yunhong Gao, Yuntao Gao, Bin Huang, Zhen Meng, Yudong Jia

**Affiliations:** 1 Yellow Sea Fisheries Research Institute, Chinese Academy of Fishery Sciences, Qingdao Key Laboratory for Marine Fish Breeding and Biotechnology, Qingdao, 266071 China; 2 College of Fisheries and Life Science, Shanghai Ocean University, Shanghai, 201306 China

**Keywords:** Molecular biology, Reproductive biology

## Abstract

Quantitative real-time reverse transcription-polymerase chain reaction (qRT-PCR) is a powerful and sensitive method used in gene expression analysis. Suitable reference genes, which are stable under all experimental circumstances and tissues significantly improve the accuracy of qRT-PCR data. In this study, the stability of six genes, namely, 18S ribosomal RNA (18*s*), beta-actin (*actb*), elongation factor 1-alpha (*ef1α*), glyceraldehyde-3-phosphate-dehydrogenase (*gapdh*), cathepsin D (*ctsd*), and beta-2-microglobulin (*b2m*) were evaluated as potential references for qRT-PCR analysis. The genes were examined in the hypothalamus-pituitary-ovary-liver (HPOL) axis throughout turbot ovarian development via using the geNorm, NormFinder and BestKeeper algorithms. Results showed that the most stable reference genes were *ef1α*, *actb*, and *ctsd* in the hypothalamus, pituitary, ovary and liver, respectively. The best-suited gene combinations for normalization were 18*s*, *ef1α*, and *ctsd* in the hypothalamus; *actb*, *ctsd*, and 18*s* in the pituitary; *actb*, and *ctsd* in the ovary; *gapdh* and *ctsd* in the liver. Moreover, the expression profile of estrogen receptor α (*erα*) manifested no significant difference normalization to the aforementioned best-suited gene during turbot ovarian development. However, no single gene or pair of genes is suitable as an internal control and account for the amplification differences among the four tissues during ovarian development. In summary, these results provide a basic data for the optimal reference gene selection and obtain highly accurate normalization of qRT-PCR data in HPOL axis-related gene expression analysis during turbot ovarian development.

## Introduction

Quantitative real-time reverse transcription-polymerase chain reaction (qRT-PCR) is widely used in gene expression analysis because of the advantages of its sensitivity and the accurate detection of mRNA at extremely low transcription levels^1^. The successful application of qRT-PCR depends on accurate transcript normalization via the selection of suitable reference genes. The reference genes minimize errors attributed to the use of biological samples, such as experimental operations, and RNA and cDNA qualities^2^. The ideal reference gene for qRT-PCR exhibits a stable expression level in all target tissues/cells and should be no affected by various experimental conditions or treatments. In general, the commonly used reference genes mainly include glyceraldehyde-3-phosphate-dehydrogenase (*gapdh*), 18S ribosomal RNA (18*s*), beta actin (*actb*), elongation factor 1-alpha (*ef1α*), cathepsin D (*ctsd*), and beta-2-microglobulin-like (*b2m*) in turbot^3,4^ and other fish species^5–8^. However, these classical reference genes vary at the transcription level in different tissues, at different developmental stages and experimental conditions or treatments^9–11^. Inappropriate reference genes that serve as internal controls can affect the accuracy of qRT-PCR results and cause the significantly different conclusion^5,12,13^. Actually, no single reference gene is universally applicable in gene expression studies. Numerous literatures have identified that two or more reference genes are generally required for qRT-PCR to minimize experimental errors caused by instability of a single gene under all experimental conditions^14–16^.

Expression stability is the most important characteristic of reference genes. Several softwares include geNorm, Normfinder and Bestkeeper are commonly used to determine optimal reference genes by calculating the stability values of these genes under different conditions^17–19^. geNorm identifies the expression stability of a candidate gene by calculate M value and pairwise variation (Vn/n + 1). NormFinder confirms the best-suited reference genes via estimating the inter and intragroup expression variations of candidate genes. BestKeeper evaluates the stability of candidate genes by calculating the standard deviation (*SD*) and correlation coefficient (*r*). In general, two or more software programs are used together during data analysis to identify discrepancies between the outputs of different algorithms^5,20,21^.

Turbot is a widely cultured economic marine fish owing to its high market value in various Asian and European countries. In our previous studies, we cloned and identified the functional properties of hypothalamus-pituitary-ovary-liver (HPOL) axis-related genes, gonadotrophin receptors and estrogen receptors (ers) during the ovarian development of turbot^22–24^. The 18*s*, actb and ctsd are the most stable housekeeping gene used for turbot luteinizing hormone receptor, ers, growth hormone and insulin-like growth factor expression analysis^22,24,25^. Thus, no single reference gene is universally applicable in turbot HPOL axis related genes expression analysis. Meanwhile, the biochemical composition of turbot eggs and ovarian fluid, that is related to egg quality was evaluated throughout the reproductive cycle^26–28^. To further investigate and fully understand the endocrine, paracrine and autocrine mechanisms during the reproductive cycle of female turbot, we need to select the new or different suitable reference genes for HPOL axis-related genes functions evaluation during ovarian development. Therefore, we aimed to investigate appropriate reference gene used for transcriptional expression analysis in turbot hypothalamus, pituitary, ovary and liver during ovarian development. The six reference genes including, 18*s*, bact, gapdh, ef1α, ctsd, and b2m were selected and expression stability through ovarian development were analyzed via using geNorm, NormFinder and BestKeeper algorithms, respectively. Previous study has identified that estrogen receptor α (ERα) is a subtype of estrogen receptors that is widely expressed in all tissues and has high mRNA levels in the hypothalamus, pituitary, ovary and liver of turbot^22^. To further confirm the ideal reference genes and investigate the function of HPOL axis-related genes. Thus, we use erα mRNA expression as an example to evaluate the effect of the selected reference genes on data normalization. The stability analysis results will provide helpful guidelines for optimal reference gene selection and make it possible to obtain more reliable results of target gene expression during turbot ovarian development.

## Materials and Methods

### Animal management and sampling

**Figure 1:**
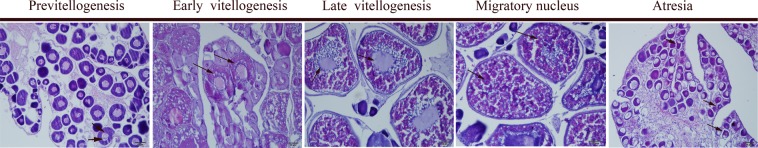
Development and types of turbot oocyte in ovarian stage. Previtellogenesis, arrow indicates nucleoli at the periphery of the germinal vesicle. Early vitellogenesis, arrow indicates gradually accumulations of yolk granules in the central region of the oocyte. Late vitellogenesis, arrow indicates the yolk granules almost fill the ooplasm, and the nucleus has not yet begun to migrate peripherally. Migratory nucleus, arrow indicates the oocytes, and the yolk granules have attained their maximum size just prior to spawning, and the nucleus is not evident. Atresia, arrow indicates the oocytes have shrinkage or collapse.

Female turbots were collected from Tianyuan Aquaculture Co., Ltd. of Yantai, China. Fishes were reared in polyethylene tanks and fed with the basal diet for one week to acclimate to the environmental conditions based on our previous study^23^. The fishes were fasted for 24 h and anesthetized with 100 mg/L tricaine methane sulfonate (MS-222, Sigma, St. Louis, MO) before sampling. Subsequently, hypothalamus, pituitary, liver, and ovary were collected from each fish and stored in liquid nitrogen for RNA extraction. Meanwhile, the ovaries were fixed in Bouin’s solution for histology. After cutting ovarian sections and mounting on glass slides, the sections were stained with hematoxylin and eosin for the identification of the ovary developmental stages based on our previous study^23^. The ovary developmental stages were classified into pre-vitellogenesis (Prevtg), early vitellogenesis (Evtg), late vitellogenesis (Latvtg), migratory-nucleus (Mig-nucl), atresia (Atre) and described in Fig. 1. All sampling procedures and subsequent experimental protocol were conducted and approved in accordance with the guidelines established by the Institutional Animal Care and Use Committee at Yellow Sea Fisheries Research Institute.

### RNA extraction and cDNA synthesis

Total RNA was extracted using a MiniBEST Universal RNA extraction kit (Takara Biotech, China) based on the manufacturer’s instructions. The yield and purity of total RNA were determined at 260 nm (A260) absorbance and 260/280 nm (A260/280) ratio by using NanoDrop ND-2000 spectrophotometer (Thermo Fisher Scientific). The RNA integrity and RNA integrity number (RIN) were assessed by agarose gel and 2100 Bioanalyzer (Agilent Technologies), respectively. The RIN is a number on a scale from 1 to 10, value of 10 indicates intact and non-fragmented RNA, and value of 1 represents completely degraded RNA^29^. In addition, the total RNA was treated with DNase I (Qiagen) for 30 min at 37 °C to avoid contamination of genomic DNA. Meanwhile, a negative control without cDNA was also included ensure that the reagents were not contaminated. Total RNA (1 μg) from each sample was reverse transcribed via a PrimeScript^TM^ RT reagent kit with cDNA Eraser (Takara Biotech, China). The resulting cDNA was diluted (1:10) with sterile deionized water and used in qRT-PCR via ABI 7900HT thermocycler (Applied Biosystems).

### Primer design and PCR efficiency

Six reference genes namely, *gapdh*, 18*s*, *actb*, *ef1a*, *ctsd*, *b*2*m* and one target gene *erα* were used in this study. The full gene names, functions, and accession numbers were listed in Table 1. Primer pairs were designed with Primer 5 software and synthesized by Sangon Biotech (Shanghai) Co., Ltd., China. The cycling conditions for the PCR reaction were 95 °C for 5 min, followed by 35 cycles of 94 °C for 1 min, 60 °C for 30 s, and 72 °C for 30 s, and a final extension of 72 °C for 5 min. The PCR efficiencies (*E*) and coefficient of determination (*r*^2^) were established on the basis of the slopes of the standard curves generated from a 10-fold dilution series of purified PCR fragments (1:10,000 dilution) as templates. *E* was calculated using the formula *E* (%) = (10^−1/slope^−1) × 100, and values between 90% and 110% were considered acceptable. All primers information about *E*, *r*^*2*^ and product lengths are listed in Table 2.

**Table 1:** Candidate reference genes used in this study.

Abbreviation	Reference gene name	Gene function	Accession number
GAPDH	*Glyceraldehyde-3-phosphate-dehydrogenase*	Glycolysis enzyme	DQ848904
18S	*18S ribosomal RNA*	Ribosomal subunits	EF126038
ACTB	*Beta actin*	Cytoskeletal structural protein	AY008305
EF1A	*Elongation factor 1-alpha*	Translational elongation	AF467776
CTSD	*Cathepsin D*	Endoproteolytic aspartic proteinase	EU077233
B2M	*Beta-*2*-microglobulin-like*	Cytoskeletal protein	DQ848854

**Table 2:** Primers and related information of the candidate reference genes and the target gene.

Gene	Primer pairs (5′-3′)	Efficiency (%)	Coefficient of determination (*r*^*2*^)	product size (bp)
GAPDH	F: GTATTGGCCGTCTGGTCCT R: GGGAGACCTCACCGTTGTAA	98.0	0.997	144
18S	F: GTGGAGCGATTTGTCTGGTT R: CTCAATCTCGTGTGGCTGAA	96.5	1.000	130
ACTB	F: CATGTACGTTGCCATCCAAG R: ACCAGAGGCATACAGGGACA	104.9	0.996	138
EF1A	F: CGGCCACCTGATCTACAAGT R: GCCTTCAGTTTGTCCAGCA	90.9	0.997	123
CTSD	F: GAAGAAGGTGGAGCAGAACG R: TGCGGGTGATGTTGATGTAG	96.8	0.999	137
B2M	F: GGCAGTTCCATCTGACCAAG R: ATGTTTGACTCCCAGGCGTA	92.8	0.998	112
ERα	F: GCCACCACTATCTGGAAACC R: CCTGACTCCCCCAAACTGTA	90.9	0.996	115

### Quantitative real time RT-PCR

qRT-PCR was performed in triplicate using ABI 7900HT thermocycler (Applied BioSystems, USA) according to the manufacturer’s instructions. Total reaction volume was 20 μL, including 2 μL of the cDNA sample, 10 μL of SYBR^®^ Primix Ex Taq II (Takara Biotech, China), 0.4 μL of ROX Reference Dye (50×), 0.8 μL of the forward/reverse PCR primers (10 μM), and 6 μL of nuclease-free water. The reaction program was 95 °C for 30 s, followed by 40 cycles of 95 °C for 10 s and 60 °C for 30 s. A negative control without cDNA was included in each assay. Melt curves were obtained by increasing the temperature from 60 °C to 95 °C at increments of 0.5 °C to confirm that only one product was amplified. The amplicons were run on a 3% high resolution buffered agarose gel with a 50 bp ladder (50, 100, 150, 200, 300, 400 and 500) and visualized with ethidium bromide (1 × TAE buffer at 90 V for 1 hour).

### Statistical analysis

The stability of Ct values was analyzed via geNorm, NormFinder and BestKeeper software. One-way ANOVA was conducted using SPSS 16.0 software (SPSS Inc., USA). The level of significance was chosen at *P* < 0.05 and Duncan’s test was conducted when necessary for multiple comparisons. Results were expressed as mean ± SEM (standard error of the mean).

## Results

### qRT-PCR amplification of candidate reference genes

The yield, purity and integrity of total RNA totally qualify for the experimental requirements (Supplemental data, S1,S2). The six reference genes, namely, *gapdh*, 18*s*, *actb*, *ef1a*, *ctsd*, and *b*2*m* were amplified from the target samples via qRT-PCR. Melting curve analysis was performed after thermocycling to determine the specifics of the PCR amplifications. The *E* values of the six candidate reference genes ranged from 92.8% to 104.9% and the *r*^*2*^ of the standard curves ranged from 0.996 to 1 (Table 2). The negative control without cDNA manifest no amplicons on the gel (Supplemental data, S3). The reference genes were reliably amplified and checked via agarose gel electrophoresis (Supplemental data, S4).

### C_t_ values of the reference genes in HPOL tissues during turbot ovarian development

**Figure 2:**
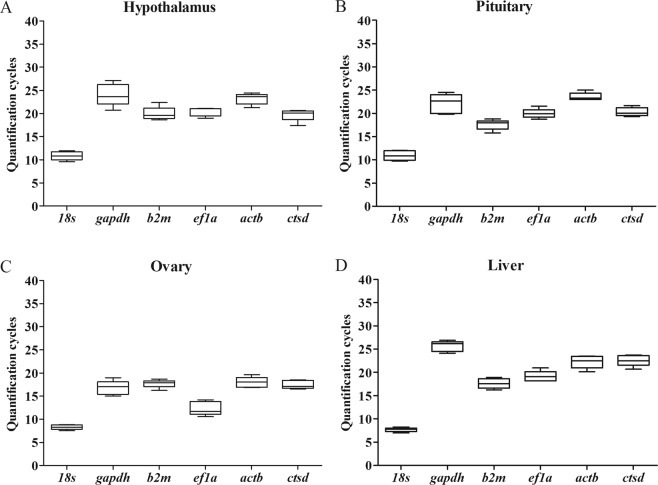
The range of expression of the candidate reference genes in hypothalamus, pituitary, ovary and liver of female turbot. The horizontal line in the box plots represents the median. The lower and upper boundaries of the box represent the 25th and 75th percentiles, respectively. The whiskers represent the minimum and maximum data points.

The Ct is defined as the number of cycles required for the fluorescent signal to cross the threshold. C_t_ levels are inversely proportional to the amount of target nucleic acid in the sample, low values indicate a high target amount, and high values indicate the opposite^30^. During the turbot ovarian development, the expression of the six reference genes varied in tissue-dependent manner (Fig. 2). 18*s* and *actb* showed the lowest C_t_ variation in the hypothalamus (0.65) and pituitary (0.60), respectively, whereas *gapdh* exhibited the highest C_t_ variation in the hypothalamus (1.78) and pituitary (1.74). The 18*s* showed the lowest level of changes in expression in the ovary (0.40) and liver (0.32), whereas *ef1a* showed the highest change in expression in the ovary (1.24) and liver (1.20). The average C_t_ values of the six reference genes in the hypothalamus, pituitary, ovary, and liver throughout ovarian development are included in Table 3.

**Table 3:** Ct values of candidate reference genes expressed in hypothalamus, pituitary, ovary and liver during ovarian development of turbot.

Tissues	Stages	GAPDH	18S	ACTB	EF1A	CTSD	B2M
Hypothalamus	Prevtg	23.30	9.72	21.30	19.03	17.45	22.37
	Evtg	20.76	10.89	22.86	21.05	20.47	19.98
	Latvtg	27.14	10.48	23.84	19.97	19.98	19.65
	Mig-nucle	23.68	11.95	24.45	21.08	20.10	19.15
	Atre	25.44	11.5	23.69	21.05	20.65	18.65
Pituitary	Prevtg	24.46	10.12	23.01	19.60	19.71	18.00
	Evtg	22.68	12.02	23.71	21.55	20.09	17.46
	Latvtg	20.10	10.85	23.26	20.03	20.74	19.91
	Mig-nucle	23.57	11.91	24.99	19.88	21.67	19.75
	Atre	19.80	9.73	23.00	18.70	19.27	15.77
Ovary	Prevtg	18.98	8.85	18.04	11.57	16.95	16.24
	Evtg	15.03	8.09	16.87	11.71	16.50	17.98
	Latvtg	15.66	8.70	17.06	10.56	17.07	17.89
	Mig-nucle	17.00	8.31	18.44	13.49	18.56	17.81
	Atre	17.18	9.57	19.62	14.22	18.35	18.70
Liver	Prevtg	26.91	7.86	21.74	18.18	22.55	17.57
	Evtg	26.18	7.00	20.15	19.27	23.45	18.37
	Latvtg	24.17	7.75	22.53	20.97	20.70	16.96
	Mig-nucle	26.31	8.26	23.44	18.92	23.76	18.92
	Atre	24.82	7.57	23.50	16.26	22.41	16.26

Prevtg: previtellogenesis; Evtg: early vitellogenesis; Latvtg: late vitellogenesis; Mig-nucle: migratory nucleus; Atre: atresia.

### Expression stability of candidate reference genes analyzed by geNorm

**Figure 3:**
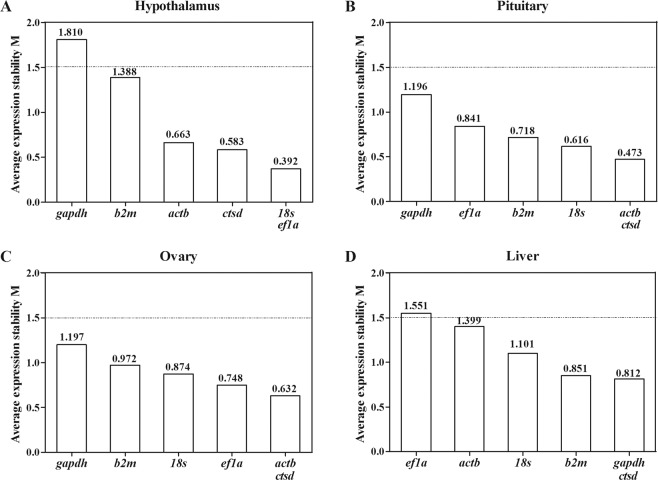
Gene expression stability and ranking orders of the candidate reference genes in hypothalamus (**A**), pituitary (**B**), ovary (**C**), and liver (**D**) during ovarian development of turbot analyzed by geNorm.

geNorm was used to calculate the expression stability index (M) of the six reference genes through the pairwise comparison of variations in expression ratios. A low value of M indicates a stable gene expression. In general, genes with an M value above 1.5 were not considered to be stably expressed. In the hypothalamus, 18*s* and *ef1a* exhibited the lowest M values and were therefore considered the most stable genes, followed by *ctsd*, *actb*, and *b2m* in terms of decreasing stability (Fig. 3A). However, *gapdh* was the least stable with M value of 1.810 in the hypothalamus (Fig. 3A). In the pituitary, *actb* and *ctsd* were ranked as the most stable genes, followed in decreasing stability by 18*s*, *b2m*, *ef1a*, and *gapdh* (Fig. 3B). In the ovary, *actb* and *ctsd* were the most stable genes, and *ef1a*, 18*s*, *b2m* and *gapdh* showed decreasing stability (Fig. 3C). In the liver, *ctsd* and *gapdh* were considered as the most stable genes, whereas *ef1a* appeared to be the least stable with an M value of 1.551 (Fig. 3D).

**Figure 4:**
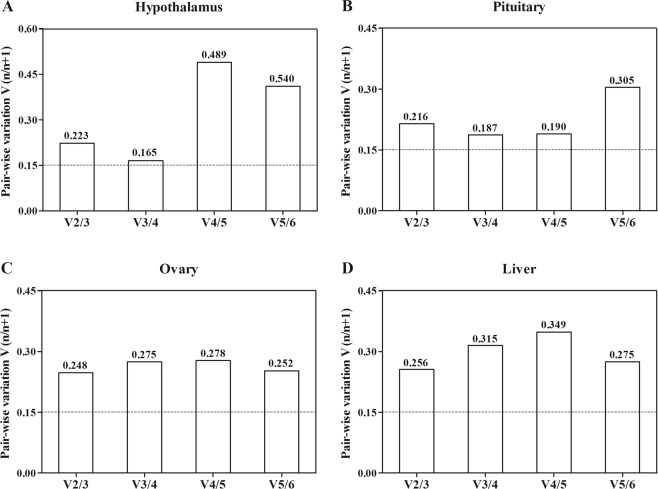
The number of the candidate reference genes required for accurate normalization in hypothalamus (**A**), pituitary (**B**), ovary (**C**), and liver (**D**) during ovarian development of turbot determined by geNorm.

The optimal number of genes required for data normalization was also analyzed using geNorm through the calculation of pairwise variations (V_n/n+1_) between sequential normalization factors n and n + 1. A threshold value of 0.15 has been suggested as the variation below which the inclusion of additional genes is not required for normalization. In the current study, all of the V_n/n+1_ values obtained were higher than 0.15. The lowest V_n/n+1_ values were 0.165 (V_3/4_) in the hypothalamus (Fig. 4A), 0.187 (V_3/4_) in the pituitary (Fig. 4B), 0.248 (V_2/3_) in the ovary (Fig. 4C), and 0.187 (V_2/3_) in the liver (Fig. 4D).

Considering the M and V_n/n+1_ values, the optimal reference genes for accurate normalization were three genes (18*s*, *ef1a*, *ctsd*) in the hypothalamus, three genes (*actb*, *ctsd*, 18*s*) in the pituitary, two genes (*actb*, *ctsd*) in the ovary, and two genes (*gapdh*, *ctsd*) in the liver.

### Expression stability of candidate reference genes analyzed by NormFinder

**Figure 5:**
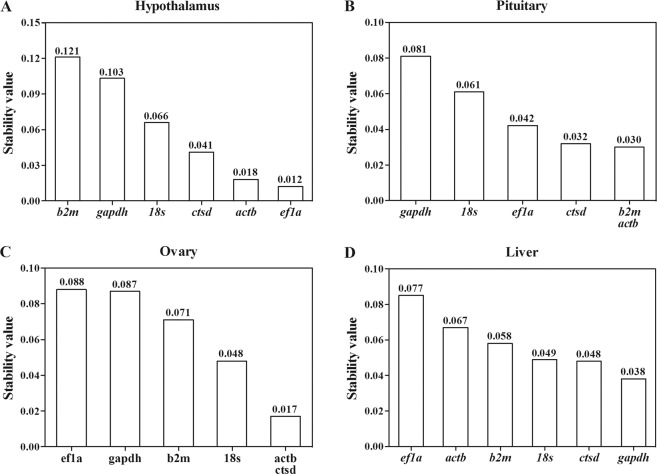
Gene expression stability and ranking orders of the candidate reference genes in hypothalamus (**A**), pituitary (**B**), ovary (**C**), and liver (**D**) during ovarian development of turbot determined by Normfinder.

NormFinder was used to estimate and combine the intergroup and intragroup expression variations of reference genes and ultimately obtain a stability value. A low value, equated to a stable gene expression. In the hypothalamus, the stability ranking was *ef1a* > *actb* > *ctsd* > 18*s* > *gapdh* > *b2m* (Fig. 5A). In the pituitary, the stability ranking was *b2m* = *actb* > *ctsd* > *ef1a* > 18*s* > *gaph* (Fig. 5B). In the ovary, the stability ranking was *actb* = *ctsd* > 18*s* > *b2m* > *gapdh* > *ef1a* (Fig. 5C). In the liver, the stability ranking was *gapdh* > *ctsd* > 18*s* > *b2m* > *actb* > *ef1a* (Fig. 5D).

### Expression stability of candidate reference genes analyzed by BestKeeper

BestKeeper was used to calculate the standard deviation (SD) and correlation coefficient (*r*) of the six reference genes (Table 4). Only genes with SD < 0.95 were included into the calculation of *r*. High values of *r* (close to 1.0) indicated stably expressed genes. In the hypothalamus, 18*s* exhibited the highest *r* value and was ranked as the most stable gene. 18*s* was followed in decreasing stability by *ef1a*, *ctsd*, and *actb*. In the pituitary, 18*s* was also considered as the most stable gene, followed by *ctsd* and *actb*. In the ovary, BestKeeper ranked *actb* and *ctsd* as the first two stable genes similar to the calculation by geNorm and NormFinder. In the liver, *b2m* appeared to be the most stable gene, followed by *gapdh* and *ctsd* as the two most stable genes.

**Table 4:** Standard deviations (SD) and correlation coefficient (*r*) of the reference genes based on their quantification cycle values analyzed by Bestkeeper.

Tissue	Factor	18S	GAPDH	B2M	EF1A	ACTB	CTSD
Hypothalamus	SD	0.69	1.78	0.97	0.75	0.92	0.91
	*r*	0.96	—	—	0.95	0.91	0.93
Pituitary	SD	0.83	1.74	0.77	0.67	0.61	0.73
	*r*	0.95	—	0.82	0.71	0.83	0.86
Ovary	SD	0.38	1.14	0.59	1.23	0.84	0.78
	*r*	0.79	—	0.58	—	0.93	0.83
Liver	SD	0.28	0.94	0.82	0.78	1.06	0.82
	*r*	0.48	0.71	0.94	0.60	—	0.71

Note: Standard deviations above 0.95 are grayed and discarded from the calculation of correlation coefficient. Lower values of correlation coefficient indicate least stably expressed genes, and higher values of correlation coefficient indicate more stably expressed genes.

### Effect of normalization on ERα using different reference genes

**Figure 6:**
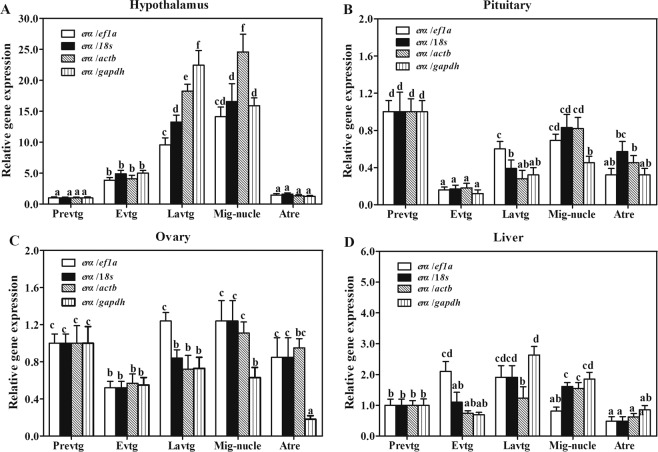
Relative quantification of the target gene *erα* in hypothalamus (**A**), pituitary (**B**), ovary (**C**), and liver (**D**) during ovarian development of turbot using different reference genes. Prevtg: previtellogenesis; Evtg: early vitellogenesis; Latvtg: late vitellogenesis; Mig-nucle: migratory nucleus; Atre: atresia. Data are presented as means ± SEM. Bars with different superscripts are statistically different (P < 0.05, n = 3).

The expression of *erα* mRNAs levels were normalized to *ef1a*, 18*s*, *actb*, *gapdh* in the turbot hypothalamus, pituitary, ovary and liver during ovarian development. The expression profiles of *erα* in the hypothalamus were consistent when *ef1a* was used as reference controls, but showed different results when 18*s*, *actb*, and *gapdh* were used as the reference genes at the Latvtg and Mig-nucle stages (Fig. 6A). Similar results were observed *er* expression in the pituitary and ovary when *actb* or 18*s* was used as the reference controls (Fig. 6B,C). However, the expression profiles of hepatic *erα* were consistent when using *gapdh* or 18*s* as reference controls, but different with the result using *actb* or *ef1a* as reference gene at evtg, lavtg and mig-nucle stages (Fig. 6D).

## Discussion

Relative quantification is widely used in analyzing target gene transcription levels on the basis of normalization with suitable reference genes. Selecting one or more appropriate reference genes as internal controls for data normalization is necessary because universal reference genes do not exist that have constant expression. In general, the mRNA levels of target genes are usually analyzed with two or more internal controls^12,31^. Numerous studies have clearly demonstrated that reference genes are tissue-specific, conditional, and development-dependent^10,11,31^. Robledo *et al*.^4^ (2014) recommended ubiquitin (*ub*) and ribosomal protein S4 (*rps4*) for the normalization of gonadal development in turbot samples (30, 45, 60, 75, 90, 105, 120, 135 days post fertilization). Our preliminary experiment showed that the Ct values of *ub* and *rps4* significantly varied during turbot ovarian development and manifested unstable reference genes analyzed by geNorm, BestKeeper and NormFinder (Supplemental data, S5,S6). Thus, we do not include the two genes in the current study. Meanwhile, *actb* was regarded as an appropriate internal standard for the normalization of the immune-relevant genes of juvenile turbot^3^. The C_t_ values of the selected six reference genes indicate abundant target nucleic acid in the specimen, while 18*s* had the lowest C_t_ value. In the present study, the stability rankings performed by geNorm and NormFinder were more similar than the rankings calculated by BestKeeper, especially in terms of the determination of the most and least stable genes. Such difference is probably due to the fact that geNorm and NormFinder evaluating the stability of reference genes according to the variation of C_t_ values and BestKeeper calculating stability values according to the correlation coefficient of C_t_ values. The similar results of reference genes stability were reported in chicken four tissues^31^.

The ovary is an HPOL axis target organ that displays morphological and functional differences during different developmental stages^32^. Numerous studies have attempted to validate the expression stability of reference genes in the ovaries of vertebrates, including fish species. Moreover, *actb*, *ctsd*, *ef1α*, and cathepsin Z were regarded as the most stable gene at the maturation stages of the ovary in tilapia^5^. In the current study *actb* and *ctsd* were the two most suitable gene combinations for normalization in the ovary of turbot during development stages. Furthermore, using the best-suited gene can produce the similar expression profiles of *erα* during turbot ovarian development. The significantly difference of *erα* expression occurred at the lavtg and mig-nucle stages via using different reference genes in the current study. The lavtg and mig-nucle stages are the key steps during oocyte maturation in teleosts, and some reference genes may be involved in the regulation of oocyte maturation. It has identified ctsd is a lysosomal enzyme responsible for the limited cleavage of the endocytosed vitellogenin for yolk protein production during ovarian follicle growth and maturation^33^. Thus, these results further confirmed that the selection of suitable reference genes for data normalization in turbot is necessary.

The hypothalamus, pituitary, and liver are important organs that play key roles in regulating the development and maturation of the ovary in teleost. The selection of suitable reference genes for normalization in these tissues has been rarely explored. In the present study, *ef1α*/18*s*/*ctsd*, *actb*/*ctsd*, and *gapdh*/*ctsd* were the most suitable in the hypothalamus, pituitary, and liver of female turbot during ovarian development, respectively. No gene or pair of genes is actually suitable as an internal control to account for amplification differences among tissues. To find an internal standard for the cross-tissue type analysis of gene expression Gilsbach *et al*. (2006), and Nagler *et al*. (2007, 2012) added enhanced green fluorescent protein (eGFP) in *vitro* transcribed RNA to the total RNA before cDNA synthesis^34–36^. The in *vitro* reference gene (eGFP) could be consistently amplified in all samples. The target gene was then normalized to eGFP by dividing the absolute value of the gene by the absolute value of eGFP.

In conclusion, the expression stability of the six candidate reference genes (18*s*, *actb*, *ef1α*, *gapdh*, *ctsd*, *b2m*) were evaluated in HPOL tissues during turbot ovarian developmental stages using qRT-PCR. The combination of three reference genes in the hypothalamus (18*s*, *ef1α*, *ctsd*) and pituitary (*actb*, *ctsd*, 18*s*), two reference genes in the ovary (*actb*, *ctsd*) and liver (*gapdh*, *ctsd*) could be used for data normalization during ovarian development. However, no gene or pair of genes is suitable as an internal control to account for amplification differences among the four tissues during turbot reproductive cycle. These findings could provide suitable reference genes for the standardization of qRT-PCR data in studies of the roles of HPOL axis during turbot ovarian development. In addition, adding an exogenous reference gene may be more appropriate than selecting an endogenous reference gene for the cross-tissue type analysis of gene expression in the future studies

## Supplementary information

Supplementary information.

## References

[1] Huggett J, Dheda K, Bustin S, Zumla A (2005). Real-time RT-PCR normalisation strategies and considerations. Gen. Immun..

[2] Bustin SA (2009). The MIQE guidelines: minimum information for publication of quantitative real-time PCR experiments. Clin. Chem..

[3] Dang W, Sun L (2011). Determination of internal controls for quantitative real time RT-PCR analysis of the effect of Edwardsiella tarda infection on gene expression in turbot (Scophthalmus maximus). Fish. Shellfish. Immun..

[4] Robledo D (2014). Analysis of qPCR reference gene stability determination methods and a practical approach for efficiency calculation on a turbot (Scophthalmus maximus) gonad dataset. BMC Genomics.

[5] Deloffre LAM, Andrade A, Filipe AI, Canario AVM (2012). Reference genes to quantify gene expression during oogenesis in a teleost fish. Gene.

[6] Wang E (2015). Evaluation and selection of appropriate reference genes for real-time quantitative PCR analysis of gene expression in Nile Tilapia (Oreochromis niloticus) during vaccination and infection. Inter. J. Mol. Sci..

[7] Shi Y, Lu J, Wang Y, Wang S (2016). Reference gene validation for quantification of gene expression during final oocyte maturation induced by diethylstilbestrol and di-(2-ethylhexyl)-phthalate in common carp. J. Enviro Sci..

[8] Dong Z (2019). Evaluation of reference genes for quantitative real-time PCR analysis of gene expression in Hainan medaka (Oryzias curvinotus). Gen. Rep..

[9] Dhorne-Pollet S, Thélie A, Pollet N (2013). Validation of novel reference genes for RT-qPCR studies of gene expression in Xenopus tropicalis during embryonic and post-embryonic development. Dev. Dynam.

[10] McCurley AT, Callard GV (2008). Characterization of housekeeping genes in zebrafish: male-female differences and effects of tissue type, developmental stage and chemical treatment. BMC Mol. Biol..

[11] Øvergård A, Nerland AH, Patel S (2010). Evaluation of potential reference genes for real time RT-PCR studies in Atlantic halibut (Hippoglossus Hippoglossus L.); during development, in tissues of healthy and NNV-injected fish, and in anterior kidney leucocytes. BMC Mol. Biol..

[12] Cao S (2012). Evaluation of putative internal reference genes for gene expression normalization in Nannochloropsis sp. by quantitative real-time RT-PCR. Biochem. Bioph Res..

[13] Fuentes EN, Safian D, Valdés JA, Molina A (2013). Isolation and selection of suitable reference genes for real-time PCR analyses in the skeletal muscle of the fine flounder in response to nutritional status: assessment and normalization of gene expression of growth-related genes. Fish. Physiol. Biochem..

[14] Ma Q, Zhuang Z, Feng W, Liu S, Tang Q (2015). Evaluation of reference genes for quantitative real-time PCR analysis of gene expression during early development processes of the tongue sole (Cynoglossus semilaevis). Acta Oceanologica Sin..

[15] Mitter K (2009). Evaluation of candidate reference genes for QPCR during ontogenesis and of immune-relevant tissues of European seabass (Dicentrarchus labrax). Comp. Biochem. Physiol. B.

[16] Yang CG (2013). Evaluation of reference genes for quantitative real-time RT-PCR analysis of gene expression in Nile tilapia (Oreochromis niloticus). Gene.

[17] Andersen CL, Jensen JL, Ørntoft TF (2004). Normalization of real-Time quantitative reverse transcription-PCR data: A model-based variance estimation approach to identify genes suited for normalization, applied to bladder and colon cancer data sets. Can. Res..

[18] Pfaffl MW, Tichopad A, Prgonmet C, Neuvians T (2004). Determination of stable housekeeping genes, differentially regulated target genes and samples integrity: BestKeeper-Excel-based tool using pair-wise correlations. Biotechnol. Let..

[19] Vandesompele J (2002). Accurate normalization of real-time quantitative RT-PCR data by geometric averaging of multiple internal control genes. Gen. Biol..

[20] Hibbelet S, Scharsack JP, Becker S (2008). Housekeeping genes for quantitative expression studies in the three-spined stickleback Gasterosteus aculeatus. BMC Mol. Biol..

[21] Zhang B, Sun L, Xiao Z, Hu Y (2014). Quantitative real time RT-PCR study of pathogen-induced gene expression in rock bream (Oplegnathus fasciatus): Internal controls for data normalization. Mar. Genomics.

[22] Hu P, Meng Z, Jia Y (2018). Molecular characterization and quantification of estrogen receptors in turbot (Scophthalmus maximus). Gen. Comp. Endocrinol..

[23] Jia Y, Meng Z, Niu H, Hu P, Lei J (2014). Molecular cloning, characterization, and expression analysis of luteinizing hormone receptor gene in turbot (Scophthalmus maximus). Fish. Physiol. Biochem..

[24] Jia Y, Sun A, Meng Z, Liu B, Lei J (2016). Molecular characterization and quantification of the follicle-stimulating hormone receptor in turbot (Scophthalmus maximus). Fish. Physiol. Biochem..

[25] Jia YD, Jing QQ, Gao Y, Huang B (2019). Involvement and expression of growth hormone/insulin-like growth factor member mRNAs in the ovarian development of turbot (Scophthalmus maximus). Fish. Physiol. Biochem..

[26] Jia Y, Meng Z, Liu X, Lei J (2014). Biochemical composition and quality of turbot (Scophthalmus maximus) eggs throughout the reproductive season. Fish. Physiol. Biochem..

[27] Jia Y, Meng Z, Liu X, Lei J (2014). Molecular components related to egg quality during the reproductive season of turbot (Scophthalmus maximus). Aqua Res..

[28] Jia YD, Niu HX, Meng Z, Liu X, Lei J (2015). Biochemical composition of the ovarian fluid and its effects on the fertilization capacity of turbot Scophthalmus maximus during the spawning season. J. Fish. Biol..

[29] Schroeder A (2006). The RIN: an RNA integrity number for assigning integrity values to RNA measurements. BMC Mol. Biol..

[30] Bubner B, Baldwin IT (2004). Use of real-time PCR for determining copy number and zygosity in transgenic plants. Plant. Cell Rep..

[31] Bagés S, Estany J, Tor M, Pena RN (2015). Investigating reference genes for quantitative real-time PCR analysis four chicken tissues. Gene.

[32] Gillies K, Krone SM, Nagler JJ, Schultz IR (2016). A computational model of the rainbow trout hypothalamus-pituitary-ovary-liver axis. PLoS Comput. Biol..

[33] Carnevali O, Cionna C, Tosti L, Lubzens E, Maradonna F (2006). Role of cathepsins in ovarian follicle growth and maturation. Gen. Comp. Endocrinol..

[34] Gilsbach R, Kouta M, Bonisch H, Bruss M (2006). Comparison of *in vitro* and *in vivo* reference genes for internal standardization of real-time PCR data. BioTec.

[35] Nagler JJ, Cavileer T, Sullivan J, Cyr DG (2007). The complete nuclear estrogen receptor family in the rainbow trout: Discovery of the novel ERα2 and both ERβ isoforms. Gene.

[36] Nagler. JJ (2012). Estrogen receptor mRNA expression patterns in the liver and ovary of female rainbow trout over a complete reproductive cycle. Gen. Comp. Endocrinol..

