# Supplementary material for: Reference gene validation for quantification of gene expression during ovarian development of turbot (Scophthalmus maximus)
Source: Sci Rep. 2020 Jan 21;10:823. doi: 10.1038/s41598-020-57633-3 (PMC6972784; doi:10.1038/s41598-020-57633-3)
Supplement: Supplementary file 1 — Supplementary information. [file 41598_2020_57633_MOESM1_ESM.pdf]

# Reference gene validation for quantification of gene expression during ovarian development of turbot (*Scophthalmus maximus*)

**Yunhong Gao[a,b] , Yuntao Gao[a,b], Bin Huang[a], Zhen Meng[a], Yudong Jia[a]***

[a] Yellow Sea Fisheries Research Institute, Chinese Academy of Fishery Sciences; Qingdao Key Laboratory for Marine Fish Breeding and Biotechnology, Qingdao 266071, China

[b] College of Fisheries and Life Science, Shanghai Ocean University, Shanghai 201306, China

* Corresponding author

**Supplementary table 1.** The yield and purity of total RNA in hypothalamus, pituitary, liver, and ovary during turbot ovarian development. Prevtg: previtellogenesis; Evtg: early vitellogenesis; Latvtg: late vitellogenesis; Mig-nucle: migratory nucleus; Atre: atresia.

| Tissues | Stages | Yield (ng/μl) | Purity (260/280) |
|---|---|---|---|
| Hypothalamus | Prevtg | 678.3 | 1.96 |
| | Evtg | 635.1 | 1.89 |
| | Latvtg | 1079.5 | 1.98 |
| | Mig-nucle | 737.5 | 1.97 |
| | Atre | 535.2 | 1.98 |
| Pituitary | Prevtg | 308.9 | 1.93 |
| | Evtg | 322.2 | 1.85 |
| | Latvtg | 273.1 | 1.95 |
| | Mig-nucle | 342.6 | 1.96 |
| | Atre | 362.5 | 1.97 |
| Ovary | Prevtg | 479.4 | 1.86 |
| | Evtg | 515.5 | 1.92 |
| | Latvtg | 466.7 | 1.98 |
| | Mig-nucle | 354.0 | 1.94 |
| | Atre | 547.2 | 1.88 |
| Liver | Prevtg | 681.3 | 1.99 |
| | Evtg | 718.9 | 2.00 |
| | Latvtg | 446.2 | 1.91 |

| | | |
|---|---|---|
| Mig-nucle | 492.6 | 1.96 |
| Atre | 822.4 | 1.97 |

**Supplementary table 2**. Standard deviations (SD) and correlation coefficient (*r*) of the *ub* and *rsp4* based on their quantification cycle values analyzed by Bestkeeper. The standard deviation (SD) > 0.95 and discarded from the calculation of correlation coefficient.

| Tissue | Factor | UB | RP4 |
|---|---|---|---|
| Hypothalamus | SD | 2.14 | 1.61 |
| | *r* | - | - |
| Pituitary | SD | 1.45 | 1.53 |
| | *r* | - | - |
| Ovary | SD | 1.49 | 1.77 |
| | *r* | - | -- |
| Liver | SD | 1.40 | 1.87 |
| | *r* | - | - |

**Supplementary Figure 1**.

a

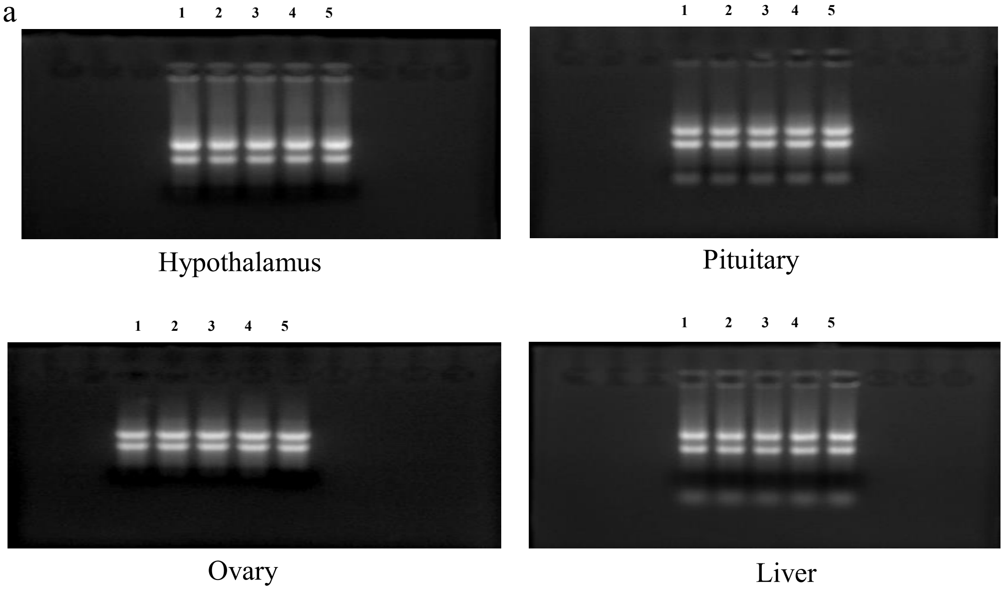


b

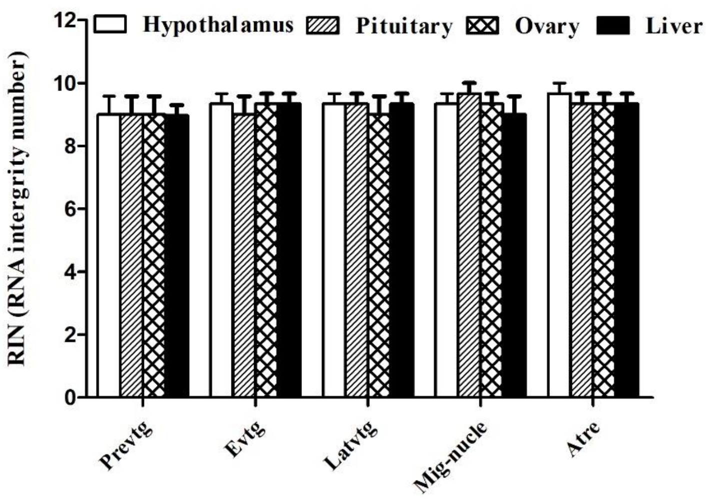


**Supplementary Figure 1**: The integrity of total RNA (a) agarose gel electrophoresis (b) RIN number in hypothalamus, pituitary, liver, and ovary during turbot ovarian development. RIN: RNA integrity number. Lanes from 1 to 5 are previtellogenesis, early vitellogenesis, late vitellogenesis, migratory nucleus and atresia stages, respectively. Other lanes were not used.

**Supplementary Figure 2**

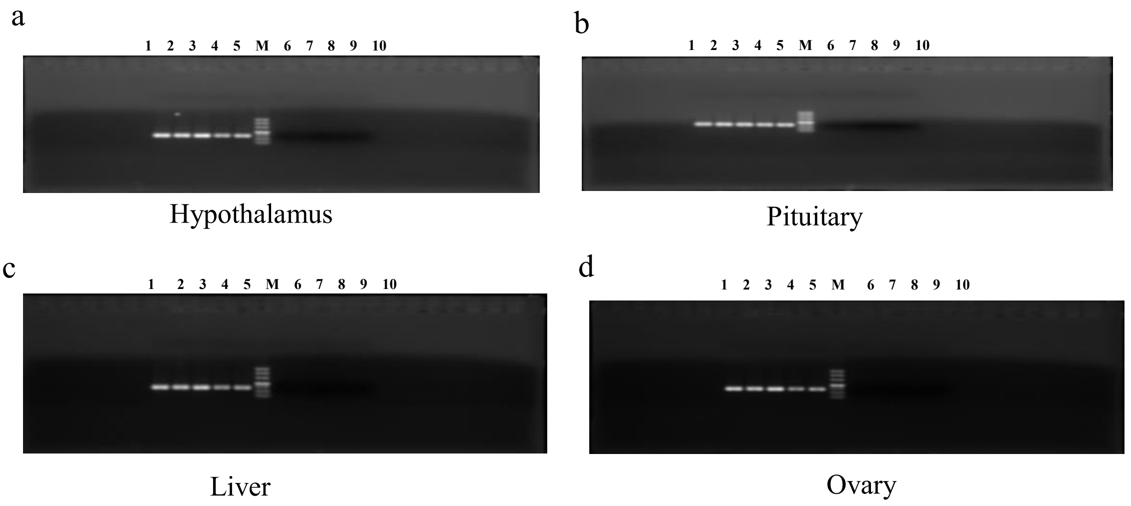


**Supplementary Figure 2**: The negative control without cDNA included in (a) hypothalamus, (b) pituitary, (c) liver, and (d) ovary during turbot ovarian development via qRT-PCR. 2% typical agarose gel stained with ethidium bromide showing the amplification of 18s. Left lanes from 1 to 5 are previtellogenesis, early vitellogenesis, late vitellogenesis, migratory nucleus and atresia stages, respectively. Right lanes from 6 to 10 are negative control. M: mark (50, 100, 150, 200, 300, 400, 500bp). Other lanes were not used.

**Supplementary Figure 3**

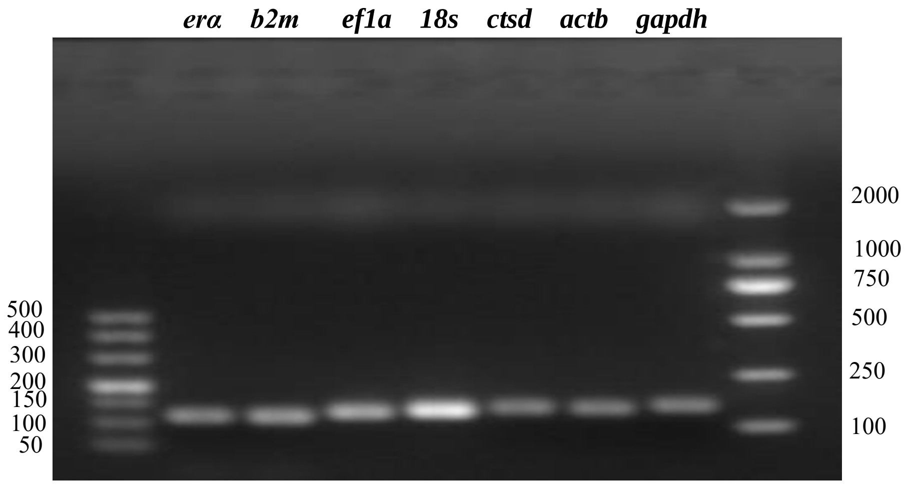


**Supplementary Figure 3**: 2% typical agarose gel stained with ethidium bromide showing the amplification of reference genes. The product size of *erα* (115bp), *b2m* (112bp), *ef1α* (123bp), *18s* (130bp), *ctsd* (137bp), *actb* (138bp) and *gapdh* (144bp) on gel via agarose gel electrophoresis.

**Supplementary Figure 4**

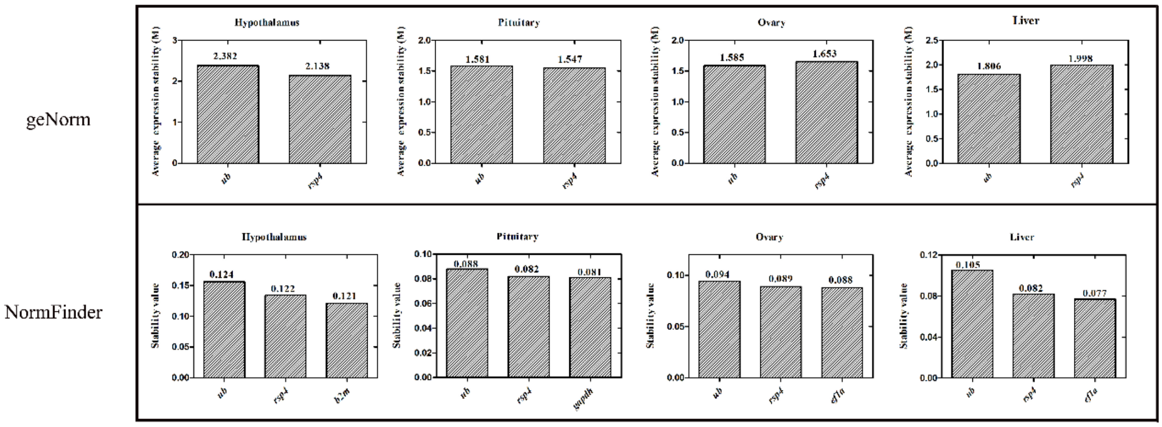


**Supplementary Figure 4:** Expression stability of *ub* and *rsp4* analyzed by geNorm and NormFinder. The M value of *ub* and *rp4* above 1.5, while exhibited the high stability value (the value negatively correlated to gene stability). Meanwhile, standard deviation (SD) > 0.95 in **Supplementary table 2** data. Thus, these two genes considered the unstable genes and not include in the current study.
